# How the Spreading and Intensity of Interictal Epileptic Activity Are Associated with Visuo-Spatial Skills in Children with Self-Limited Focal Epilepsy with Centro-Temporal Spikes

**DOI:** 10.3390/brainsci13111566

**Published:** 2023-11-08

**Authors:** Pauline Dontaine, Coralie Rouge, Charline Urbain, Sophie Galer, Romain Raffoul, Antoine Nonclercq, Dorine Van Dyck, Simon Baijot, Alec Aeby

**Affiliations:** 1 Department of Pediatric Neurology, Hopital Universitaire de Bruxelles (H.U.B.), Queen Fabiola Children’s University Hospital (HUDERF), Université Libre de Bruxelles (ULB), 1020 Brussels, Belgium; 2 Neuropsychology and Functional Neuroimaging Research Group (UR2NF), Center for Research in Cognition & Neurosciences (CRCN), ULB Neurosciences Institute, Université Libre de Bruxelles (ULB), 1050 Brussels, Belgium; 3 Laboratoire de Neuroanatomie et Neuroimagerie Translationnelles (LN2T), UNI-ULB Neurosciences Institute, Hôpital Erasme, Université Libre de Bruxelles (ULB), 1050 Brussels, Belgium; 4 Department of Neuropsychology and Speech Therapy, Queen Fabiola Children’s University Hospital (HUDERF)-Hôpital Universitaire de Bruxelles (H.U.B), Université libre de Bruxelles (ULB), 1020 Brussels, Belgium; 5 BEAMS (Bio-, Electro- And Mechanical Systems), Université Libre de Bruxelles (ULB), 1050 Brussels, Belgium

**Keywords:** IED and cognition, visuo-spatial skills, EEG score

## Abstract

This paper investigates brain–behaviour associations between interictal epileptic discharges and cognitive performance in a population of children with self-limited focal epilepsy with centro-temporal spikes (SeLECTS). Sixteen patients with SeLECTS underwent an extensive neuropsychological assessment, including verbal short-term and episodic memory, non-verbal short-term memory, attentional abilities and executive function. Two quantitative EEG indices were analysed, i.e., the Spike Wave Index (SWI) and the Spike Wave Frequency (SWF), and one qualitative EEG index, i.e., the EEG score, was used to evaluate the spreading of focal SW to other parts of the brain. We investigated associations between EEG indices and neuropsychological performance with non-parametric Spearman correlation analyses, including correction for multiple comparisons. The results showed a significant negative correlation between (i) the awake EEG score and the Block Tapping Test, a visuo-spatial short-term memory task, and (ii) the sleep SWI and the Tower of London, a visuo-spatial planning task (*p*_corr_ < 0.05). These findings suggest that, in addition to the usual quantitative EEG indices, the EEG analysis should include the qualitative EEG score evaluating the spreading of focal SW to other parts of the brain and that neuropsychological assessment should include visuo-spatial skills.

## 1. Introduction

The link between interictal epileptic discharges (IED) and cognition has mostly been studied within the framework of self-limited focal epilepsy (SeLFE), more specifically in self-limited focal epilepsy with centro-temporal spikes (SeLECTS). This frequent epileptic syndrome accounts for 6 to 7% of all childhood epilepsies and affects children with normal cerebral MRI and development prior to seizures. Age at onset is usually between 3 to 14 years. EEG usually shows characteristic triphasic high-voltage spike-and-wave complexes that are typically located in the centro-temporal area. These abnormalities usually activate in drowsiness and sleep. Typically, most patients present rare, brief, nocturnal focal seizures involving orofacial and brachial regions and normally resolved by puberty [1]. Because of infrequent seizures, typical and frequent IED and a low need for anti-epileptic drugs, SeLECTS constitute an ideal model to study IED impact on cognition. At the most severe end of the same continuum, this link between IED and cognition is illustrated by the concept of epileptic encephalopathy with continuous spike and waves during sleep (EE-CSWS), defined as severe global or task-specific cognitive regression associated with almost continuous and diffuse IED during sleep. Because this encephalopathy may arise as a complication of SeLFE, these two entities are part of the same spectrum [2,3,4]. Interestingly, cases of Landau–Kleffner syndrome (i.e., an EE-CSWS with specific language regression in the form of auditory agnosia) are reported in patients without seizure [5]. At the opposite end of the spectrum, a SeLECTS EEG pattern is more frequently present in patients diagnosed with a developmental language disorder or attention-deficit/hyperactivity disorder than in the general population [6,7,8]. This suggests a close relationship between IED and cognition rather than an impact of seizures or anti-epileptic drugs.

Children with SeLECTS are at higher risk of developing learning disorders and academic difficulties, which are for the most part reversible after epilepsy remission [9]. Despite the common assertion that SeLECTS patients display a normal-ranged intellectual quotient (IQ), it appears statistically inferior to healthy peers [10,11]. Even if mean IQ may be overestimated by excluding patients with an IQ below 80 from most studies, it seems that, through a lack of sensitivity, exclusive analysis of IQ fails to adequately apprehend cognitive impairment in these patients [9]. Through a more comprehensive neuropsychological assessment, the literature concludes that SeLECTS is associated with an elevated frequency of language deficits, behavioural disturbances, attention-deficit/hyperactivity disorder, as well as verbal and non-verbal memory, attentional processes and executive functions impairment, with no typical cognitive pattern [9].

Associations have already been shown in SeLECTS between IED intensity, defined by quantitative parameters in wake and/or sleep EEG and academic or behavioural problems [12], specific learning disorders [13], verbal memory [14,15], verbal IQ [16], word and sentence reading [16], performance in selective visual attention [17] or central information processing speed [18].

Fewer authors used qualitative parameters to measure IED intensity, such as Massa et al., who isolated five EEG and clinical criteria predictive of complicated evolution (i.e., academic and behavioural problems) in SeLECTS [19]. In 2021, a case–control study driven by our team showed that a qualitative EEG score inspired by the aforementioned Massa study and focusing on IED spreading offered better sensitivity, specificity and agreement between readers with different levels of expertise than the usual quantitative indices (i.e., Spike Wave Index and Spike Wave Frequency) to differentiate EE-CSWS from typical SeLFE [20].

While the association between SeLECTS and possible language impairment is now broadly accepted, data regarding visuo-spatial abilities are still scarce. In this matter, poorer visuo-spatial performance were reported in children with SeLECTS compared to their healthy peers [9,21,22,23]. The few studies investigating the link between these skills and IED intensity showed an association between visuo-spatial memory and sleep Spike Wave Index (SWI) or awake Spike Wave Frequency (SWF) [24,25,26].

To date, no studies have explored the link between IED and cognition in a continuous approach within a population of children, including the whole SeLECTS continuum, from learning disorders without any history of seizures to EE-CSWS. Moreover, there are no guidelines in the literature regarding the EEG index that should be used to evaluate the epileptic activity impact on cognitive performance. The objective of this study was to answer the three following questions: first, in a population of children with SeLECTS, is there an association between IED intensity and cognitive performance, evaluated through a comprehensive neuropsychological assessment? Second, can this association not only be found in sleep but also in wakefulness? Finally, could a qualitative EEG analysis, focusing on IED spreading instead of intensity, show associations between cognitive functioning and interictal epileptic activity?

## 2. Materials and Methods

This retrospective study was approved by the Ethics Committee of the Hôpital Universitaire Des Enfants Reine Fabiola (HUDERF), Brussels, Belgium (CEH 59/22).

### 2.1. Patient Selection

Patients with SeLECTS were identified from the database of patients who underwent a long-term EEG in an epilepsy investigation unit in HUDERF between 2016 and 2022.

The patients had to fulfil the following inclusion criteria: (a) a SeLECTS EEG pattern (i.e., triphasic high-amplitude spike-and-wave complexes in the centro-temporal regions, activated in drowsiness and sleep [1]); (b) age range 6 to 12 years; (c) standardised neuropsychological assessment less than six months apart from EEG; (d) normal cerebral MRI; (e) normal neurodevelopment, defined as walking independently before 18 months, first words spoken before 18 months and first sentences before 3 years of age; and (f) no history of treatment with corticosteroids before the analysed period. The fulfilment of these criteria was determined by analysing their medical file.

Patients’ files were analysed to identify the presence of a neurocognitive regression in at least two domains of development (i.e., language, behaviour, learning, memory, attention, social interactions, motor skills and global intelligence, as assessed through comprehensive neuropsychological evaluation). Language regression was defined as a regression in at least three of the language domains (i.e., phonology, morphology, syntax, semantics and pragmatics) without auditory–verbal agnosia, and frontal syndrome was defined as behavioural disturbances with a combination of attention impairment, impulsiveness, mood swings and perseveration with deficits in reasoning, thought formulation and learning strategy [27].

### 2.2. EEG Analyses

All EEG studies were carried out using 21 scalp electrodes according to the International 10–20 system, with the BrainRT system (OSG, Belgium). Extracts of 20 min wakefulness and the first 10 min of NREM sleep were created from long-term EEG studies for each patient by author P.D. These two files were randomly and blindly renamed by a medical doctor not participating in this study.

Each segment was then scored using three EEG indices: (1) the Spike Wave Index (SWI), corresponding to the percentage of spike and wave (SW) activity calculated by dividing the number of seconds demonstrating one or more spike-and-wave complexes in the 20 min awake period divided by 1200 s, or in the 10 min period of sleep divided by 600 s, multiplied by 100 to express the results as percentages; (2) the Spike Wave Frequency (SWF), corresponding to the number of spike-and-wave complexes in the first 100 s of the EEG; (3) a qualitative EEG score that was created by Aeby et al. in 2005, based on the Massa study [19], focusing on the background (normal or abnormal, i.e., intermittent slow wave focus in wakefulness and/or abnormal or absent spindles in sleep), the number of epileptic foci and their diffusion to other electrodes. Five grades are defined: grade 0 (normal EEG); grade 1 (normal background, unique focus of SW of low amplitude); grade 2 (normal background, multiple (≥2) asynchronous foci of SW of low amplitude); grade 3 (normal or abnormal background, high-amplitude SW diffusing to one hemisphere or multiple asynchronous foci of SW of high amplitude or unique focus of high amplitude SW with a mirror focus); and grade 4 (abnormal background, high-amplitude SW diffusing to more than 80% of the electrodes) [28].

Quantitative indices (i.e., SWI and SWF) were determined by a previously published automated spike detection algorithm [29,30]. Two physicians (P.D. and A.A.) independently determined the qualitative EEG score. This EEG score was analysed with the same parameters for each patient of this study: bipolar montage, time constant of 10 s, amplitude of 100 μV/cm, high band filter of 0.3 Hz and low band filter of 70 Hz.

### 2.3. Neuropsychological Testing

All of the participants underwent a comprehensive neuropsychological assessment, developed by our team in 2016 after an extensive literature review, designed to screen SeLECTS patients for cognitive complications. The tests were performed by two experienced neuropsychologists (S.G. and S.B.). The following functions were assessed: (1) verbal short-term memory, with the WISC-V Digit Span subtest, in which the child has to repeat number lists of increasing length, forward and backwards [31]; (2) verbal long-term memory, evaluated by the RLS-15, a task where the subject must memorize a list of 15 words, with 5 free recalls and a delayed recall after 20 min [32]; (3) visuo-spatial short-term memory with the Block Tapping Test, assessing the ability to reproduce visuo-spatial sequences of increasing length by touching specific blocks placed in a two-dimensional grid [33]; (4) attention through the Test of Attentional Performance, a computerized task measuring the reaction time and its variations to a visual stimulus, with (phasic alert) or without (tonic alert) an auditory warning [34]; (5) executive functions, i.e., visuo-spatial planning with the Tower of London test, in which the child has to reorganise three pearls on three sticks in a given configuration, with respect to specific rules [35], and cognitive inhibition via the Counting Stroop Test, a task where the subject has to inhibit automatic responses [35,36,37].

### 2.4. Statistics

Statistical analyses were conducted using Jamovi [38]. The relationship between cognitive performance and EEG indices was investigated with Spearman’s nonparametric rank correlation, applying Bonferroni’s multiple comparisons correction within each cognitive domain [39]. We used raw scores for all neuropsychological measurements, and age was defined as a control variable. A *p*_corr_ < 0.05 was considered statistically significant.

## 3. Results

### 3.1. Clinical Data

Among the 125 patients with a SeLECTS EEG pattern who had a long-term EEG between 2016 and 2022, 26 patients underwent a standardised neuropsychological assessment, 19 of them less than six months apart from the EEG. We excluded two patients for an abnormal neurodevelopment and one for an abnormal cerebral MRI. We finally included 16 patients. Their detailed clinical characteristics are reported in Table 1. Age at EEG was 8 ± 1.25 years. Of all the participants, 14 were male.

Among our 16 patients, two had no history of seizures. Their EEG was performed to rule out absence seizures whilst they presented with learning disabilities. Nine patients were treated with anti-seizure medication at the time of the EEG, six with one anti-epileptic drug and three with two different anti-epileptic drugs (Levetiracetam, Valproic Acid, Clobazam or Topiramate). A cognitive regression (i.e., frontal syndrome or language impairment) concordant with an EE-CSWS was reported in four patients. The duration between the EEG and the neuropsychological testing was 2 ± 1.81 months.

### 3.2. EEG Characteristics

Detailed EEG characteristics are reported in Table 2. Two patients had no IED during wakefulness, but IED were found in their sleep EEG. The following results are expressed in median and interquartile (IQR). Awake SWI was 7.5; 51.25% (median; IQR), sleep SWI was 68; 31%. Awake SWF was 12; 54.75 (median; IQR), sleep SWF was 75; 144. Awake EEG score was 1; 3 (median; IQR) and sleep EEG score was 3; 2.5. Seven patients had a left unilateral focus, three had a right unilateral focus and six had bilateral foci.

### 3.3. Relationship between IED and Neuropsychological Data

**Figure 1:**
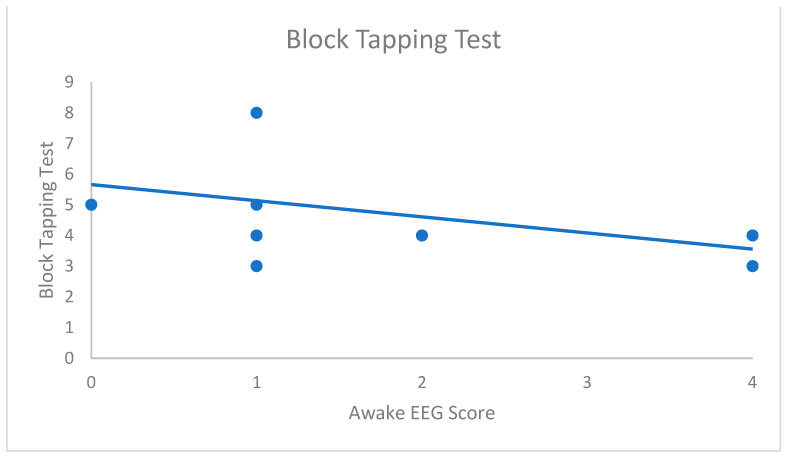
Significant association between the Block Tapping Test and the awake qualitative EEG score. (r_s_ = −0.727; *p*_corr_ < 0.05).

**Figure 2:**
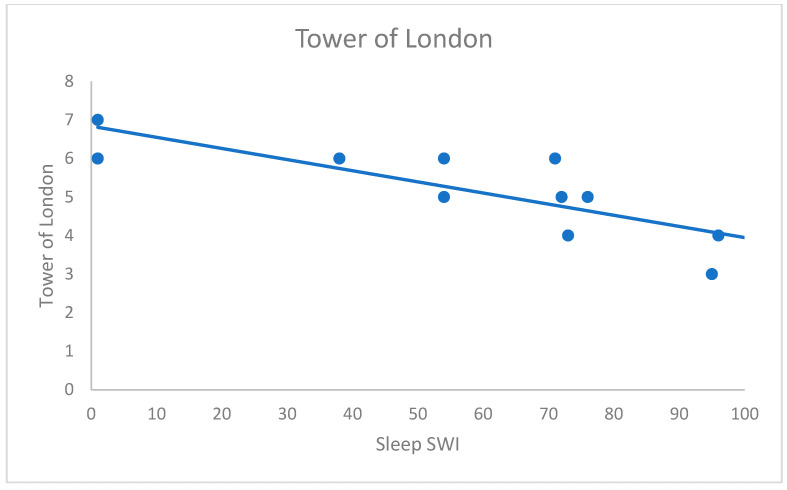
Significant association between the Tower of London and the sleep Spike Wave Index (SWI; r_s_ = −0.87; *p*_corr_ < 0.05).

The results are summarised in Table 3. Our results showed a statistically significant negative correlation between the awake EEG score and the Block Tapping Test (r_s_ = −0.73; *p*_corr_ < 0.05), highlighting that higher awake EEG score is associated with poorer performance in visuo-spatial short-term memory (Figure 1). Patients with higher sleep SWF also tended to have lower scores on the Block Tapping Test, but this tendency did not reach statistical significance after Bonferroni’s correction (r_s_ = −0.58; *p*_corr_ = 0.196). Additionally, we found a significant negative correlation between sleep SWI and the Tower of London (r_s_ = −0.87, *p*_corr_ < 0.05). In other terms, the higher the sleep SWI, the lower the score in this visuo-spatial planning task (Figure 2).

## 4. Discussion

This study is the first to investigate the link between cognition and IED in a population of children with an EEG pattern of SeLECTS, including patients without any history of seizures, patients with typical SeLECTS and patients with EE-CSWS, to better illustrate the continuum where these entities fall [4]. Moreover, we examined this link through a linear analysis to better capture the full SeLECTS spectrum, where most existing studies divided their samples by categories of IED intensity or cognitive complications [12,13,17,19,25,40].

Our results show a negative correlation between (1) IED spreading (i.e., spike-and-wave complexes diffusion to the other brain regions) in wakefulness and a short-term visuo-spatial memory task (Block Tapping Test) and (2) IED intensity in sleep and a task involving executive function, and more specifically, visuo-spatial planning (Tower of London). Therefore, our findings confirm the association between IED and cognition in SeLECTS, suggesting more precisely an association between IED and visuo-spatial skills in these children. Furthermore, they demonstrate that, in addition to epileptic activity intensity in sleep, SW spreading in wakefulness also correlates to cognitive performance in patients with SeLECTS.

Studies investigating the relationship between epileptic activity intensity and visuo-spatial performance in SeLFE are scarce. A negative correlation between long-term visuo-spatial memory performance and awake SWF has been reported by Vintan et al. in a group of 18 patients diagnosed with SeLECTS [26]. Zhang et al. showed in a larger group of 60 children with SeLECTS that patients with a sleep SWI > 55% had lower scores in a visuo-spatial memory test than patients with a sleep SWI < 55%. Moreover, bilateral epileptic foci were associated with worse visuo-spatial performance [25]. Unlike our study, none of these studies reported the application of a multiple comparisons correction during their statistical analyses.

There is evidence of possible visuo-spatial memory impairment in SeLECTS. For instance, Baglietto et al. and Metz-Lutz et al. reported lower scores in the Block Tapping Test in patients with SeLECTS compared to controls [21,41]. By contrast, Lindgren et al. failed to demonstrate a difference in their sample using a similar test [42]. In a study comparing 40 untreated patients with a SeLECTS EEG pattern, with or without related epilepsy, to 40 healthy participants, Weglage et al. showed poorer performance in short-term visuo-spatial memory [43]. In similar-sized samples, Northcott et al. and Volkl-Kernstock et al. reported an impairment of short- and long-term verbal and non-verbal memory in children with SeLECTS [23,44]. In 2021, a meta-analysis regarding working memory in paediatric epilepsy conducted by Poole et al. revealed impairments in the phonological loop and the visuo-spatial sketchpad in epileptic children. Unfortunately, no distinction was made between SeLECTS and other epileptic syndromes and the relationship between these cognitive impairments and IED was not investigated [45].

Galer et al. studied sleep-related consolidation of declarative memories in SeLFE through a verbal (associations of word pairs) and visuo-spatial (location of object pairs presented on a grid) long-term memory task. While their patients displayed similar performance to controls in immediate retrieval, their performance decreased significantly after a night of sleep, becoming inferior to controls. Furthermore, higher SWI during NREM sleep was associated with lower scores in the visuo-spatial task, supporting the hypothesis that IED could interfere with sleep-dependent memory consolidation [24].

Neuroimaging studies suggest that the neural correlates of visuo-spatial memory lie in the frontal and parietal cortices. Functional MRI studies identified a superior frontal–intraparietal network where brain activity, myelination and development of visuo-spatial working memory seem to be related during childhood [46,47]. Interestingly, several functional connectivity studies conducted in SeLECTS precisely showed altered brain functional connectivity in these regions, which seemed partly driven by the IED. Changes in connectivity strength in the default mode network were also reported in SeLECTS. The appropriate regulation of this resting-state network involving the praecuneus, medial prefrontal cortex and lateral parietal cortex seems to correlate with several cognitive functions including working memory and attentional processes. Here again, changes in connectivity seem linked to the IED [48]. In our study, no correlation was found between IED spreading or intensity and performance in attention tasks. This could be caused by our reduced number of patients, or by the great variability of attentional skills depending on cognitive availability.

To our knowledge, this study is the first to correlate visuo-spatial planification to interictal epileptic activity. However, impairments of executive functions such as inhibition, cognitive flexibility, or verbal fluency in SeLECTS were brought to light by previous studies [10,12,49,50]. Croona et al. showed poorer performance in the Tower of London task in 17 SeLECTS patients compared to a control group matched for age, sex and estimated intelligence [51]. Filippini et al. showed lower scores in a structured figural fluency task in SeLECTS patients compared to healthy peers [52]. In our study, only visuo-spatial planification correlated with epileptic activity intensity, and no relationship was found between IED and cognitive inhibition, which could possibly be related to the small size of our sample.

The finding that in wakefulness, the EEG index that focuses on the spreading of IED correlates with cognitive performance is in line with the results of Aeby et al. in 2021. Their results showed that this EEG score, measured in wakefulness and sleep, offered better sensitivity, specificity and agreement between readers with different levels of expertise than the usual quantitative indices (i.e., SWI and SWF) to differentiate EE-CSWS from typical SeLFE [20]. These observations could be explained by the “remote inhibition” concept, which proposes the existence of epilepsy-induced inhibition of neurons that surround or are remote from the epileptic focus but connected with it via cortico-cortical or polysynaptic pathways [53]. This concept originated from the demonstration, in FDG-PET studies, of focal hypermetabolism at the site of epileptic foci, associated with hypometabolism in remote connected brain areas, such as frontal and parietal cortices and the default mode network [54]. Therefore, we could extrapolate that the more an IED spreads to the other brain regions, the more it could disrupt cerebral connectivity, thus interfering with cognitive networks.

In sleep, the EEG index correlating with neuropsychological measurements is the SWI, in agreement with several studies reporting associations between sleep SWI and word memory retention, sentence reading, verbal IQ or behaviour disorders [14,16,18]. Without guidelines regarding a gold-standard index, a wide variety of indices are used to measure interictal epileptic activity in the literature. Hence, numerous studies used indices based on the number of IED in a specific time period (similar to SWF) instead of the number of seconds containing IED (i.e., SWI) and showed correlations between IED in sleep and language impairments, verbal memory or selective visual attention performance, or with the existence of academic or behavioural problems, correlated themselves with IQ, auditory–verbal, visuo-spatial and attentional capacities [12,13,15,17,19,40]. Although our analyses failed to identify a significant correlation between SWF and cognitive measurements, we found a negative tendency between sleep SWF and the Block Tapping Test, investigating visuo-spatial memory.

Exploring the link between cognition and interictal epileptic activity leads the way to a broad treatment issue. In EE-CSWS, several retrospective studies show that, under corticosteroids, a decrease in epileptic activity intensity correlates with improved cognitive performance and IQ [55,56]. Levetiracetam also appears effective in IED intensity as well as on cognitive capacities [28]. In a pilot study regarding sleep-related declarative memory consolidation in SeLFE led by Urbain et al., normalisation of the sleep EEG in an EE-CSWS patient under hydrocortisone treatment was associated with the normalisation of overnight memory performance, which was not the case in another patient under the same treatment, whose sleep EEG was only partially improved [57]. However, cognitive regression or stagnation can be difficult to identify and, because SeLECTS and EE-CSWS belong to the same spectrum, the threshold for which treatment is appropriate may be debatable. The potential benefit of anti-epileptic drugs has not yet been demonstrated in SeLECTS without cognitive regression. Even though treatment with Levetiracetam or Sulthiame seems to lead to a substantial reduction in IED intensity, it is unclear whether that decrease is associated with a cognitive improvement and this question needs to be addressed by well-designed randomized control studies [58]. A few uncontrolled studies suggest a positive impact of Levetiracetam on cognitive functions [59,60]. Amongst them, McNally et al. reported on seven patients with learning disorders and displaying a SeLECTS EEG pattern without a history of seizures and demonstrated that their parents noticed an improvement in scholarly results and/or language after the empirical introduction of Levetiracetam [61]. Nevertheless, the evidence of a visuo-spatial impairment in SeLECTS brings to light the importance of a comprehensive neuropsychological assessment in the follow-up of these patients, allowing for an early detection of such cognitive deficits, in order to implement rehabilitation strategies such as cognitive rehabilitation and individualised school care.

The main limitation of our study is the limited number of patients. Although this strengthens the significance of our results, we could have possibly brought other significant correlations to light with a larger sample. Furthermore, it could be interesting to compare our patient results with a control group of healthy peers, to better apprehend their cognitive deficits in contrast to healthy children. A certain degree of heterogeneity can also be highlighted in our sample regarding anti-epileptic drugs, a common limitation in the scientific literature [10]. It should be noted that, while the literature suggests a possible detrimental effect of Levetiracetam on behaviour, there is no consistent evidence of cognitive impairment associated with the use of Levetiracetam in children [60]. One controlled study comparing Levetiracetam and Sulthiame in a group of 80 patients with SeLECTS treated for 6 months and evaluated with a longitudinal neuropsychological assessment concluded that anti-epileptic monotherapy did not negatively affect cognitive performance. However, the impact on behaviour was more debatable, with five dropouts due to behavioural problems in the levetiracetam group, even though the study showed a tendency to a decreased CBCL total score after 6 months in both groups, indicating a reduced incidence of behavioural disturbances [62]. By contrast, Valproic Acid, in comparison to other anti-seizure medications, may be associated with an increased risk of impairments in memory, attention and executive function [60]. However, it is worth noticing that almost half of our patients were not under any treatment at the time of their EEG. This heterogeneity is also found in the neuropsychological assessment, which can be explained by the children’s cognitive availability to respond to certain tests. Because of the retrospective design, some delay can also be noted between this assessment and the EEG of each child. A shorter interval between both exams would allow us to be more representative of the children’s functioning at a given time and, consequently, to discover more associations between the children’s cognitive functioning and their epileptic activity. Moreover, in sleep, EEG indices were computed using the first ten minutes of NREM sleep, and we could have possibly brought to light a stronger relationship between IED in sleep and cognition with an entire night analysis. However, other studies have suggested that the first ten minutes of sleep are adequately representative of epileptic activity during a whole night [14]. Another limitation of our study is that, for practical reasons, the majority of our patients could not be tested for language. Therefore, the absence of an association between epileptic activity and verbal memory in our study should be interpretated with caution. Lastly, our study was conducted in a tertiary centre, which might generate a selection bias.

To better apprehend the impact of epileptic activity on cognition, further studies should investigate associations between IED intensity and spreading and performance in a comprehensive neuropsychological and language assessment in a large group of patients displaying a SeLFE EEG pattern without any history of seizures nor anti-epileptic therapy.

## 5. Conclusions

This retrospective study conducted in a population representative of the SeLECTS spectrum, from learning disorders with IED and typical SeLECTS to EE-CSWS showed an association between IED and cognition, reflecting differently during wakefulness and sleep. During sleep, we showed a negative correlation between cognitive performance and SW intensity, an association already suggested in the literature, while in wakefulness, the parameter having the most significant impact on cognitive function appears to be their spreading to other parts of the brain. Therefore, it would be useful to add an EEG score investigating wake- and sleep-related qualitative parameters in the EEG evaluation of SeLECTS patients in further studies as well as in the day-to-day care of these children. Lastly, as both cognitive functions correlating with IED were visuo-spatial, our results bring to light the relevance of comprehensive neuropsychological assessment, including visuo-spatial skills. Further studies are needed to confirm our results in a larger population.

## Figures and Tables

**Table 1:** Clinical characteristics.

Patient Number	Sex	Age at Seizure Onset (Years)	Age at the Time of EEG (Years)	Treatment during EEG	Type of Regression if Present
1	M	6	7	/	/
2	M	4	6	LVT	Frontal syndrome, language
3	M	3	6	LVT	/
4	M	4	6	LVT	Frontal syndrome, language
5	M	6	7	/	/
6	M	8	9	LVT, VPA	/
7	M	/	8	/	/
8	M	6	7	VPA	/
9	M	/	8	/	/
10	M	9	9	VPA	/
11	M	9	9	/	/
12	M	6	7	LVT, CLB	Frontal syndrome
13	F	10	10	/	/
14	F	4	7	/	/
15	M	8	9	VPA	/
16	M	7	8	VPA, TPA	Frontal syndrome

Abbreviations: CLB = Clobazam, LVT = Levetiracetam, TPA = Topiramate, VPA = Valproic Acid.

**Table 2:** EEG data.

Patient Number	Awake SWI	Awake SWF	Awake EEG Score	Sleep SWI	Sleep SWF	Sleep EEG Score	EEG Lateralisation of Interictal Foci	EEG Lobar Distribution of Interictal Foci
1	51	56	1	73	52	4	L	CT
2	98	217	4	100	352	4	B	CP, F
3	3	30	1	50	72	1	L	C
4	63	57	1	84	140	3	R	CT
5	7	9	4	54	63	4	B	F
6	87	227	4	71	284	4	B	PT
7	34	38	1	66	79	1	L	CT
8	0	0	0	1	2	2	L	C
9	12	15	2	38	49	2	B	C
10	4	2	4	76	99	4	R	F
11	7	5	1	54	49	2	B	CT
12	82	145	4	95	330	4	R	CT
13	0	2	1	1	2	1	L	F
14	0	0	0	1	2	1	L	CT
15	2	0	1	72	75	3	L	CT
16	8	0	4	96	246	4	R	CP, T

Abbreviations: SWI = Spike Wave Index, SWF = Spike Wave Frequency, R = right, L = left, B = both, C = central, CT = centro-temporal, CP = centro-parietal, F = frontal, PT = parieto-temporal, T = temporal.

**Table 3:** Spearman’s correlations (without correction) between EEG indices and neuropsychological parameters.

Field	Neuropsychological Test		Awake SWI	Awake SWF	Awake EEG Score	Sleep SWI	Sleep SWF	Sleep EEG Score
Verbal memory	Forward verbal span *n* = 12	Spearman’s Rho *p* value	0.87 0.05	0.14 0.68	−0.03 0.93	−0.43 0.16	−0.13 0.7	−0.36 0.28
	Reverse verbal span *n* = 12	Spearman’s Rho *p* value	−0.13 0.70	0 1	−0.28 0.39	−0.48 0.11	−0.39 0.23	−0.42 0.19
	RLS free recall mean *n* = 11	Spearman’s Rho *p* value	0.10 0.79	0.26 0.5	−0.19 0.62	−0.41 0.23	−0.13 0.73	−0.38 0.31
	RLS delayed recall *n* = 11	Spearman’s Rho *p* value	−0.09 0.82	0.10 0.79	−0.23 0.55	−0.51 0.13	−0.45 0.22	−0.07 0.85
Visuo-spatial memory	Block Tapping Test *n* = 13	Spearman’s Rho *p* value	−0.38 0.22	−0.42 0.18	−0.73 0.007 *	−0.45 0.12	−0.58 0.05	−0.51 0.09
Attention	Reaction time tonic alert *n* = 15	Spearman’s Rho *p* value	−0.07 0.81	−0.31 0.27	0.29 0.31	0.31 0.26	0.25 0.39	0.38 0.19
	Standard deviation tonic alert *n* = 15	Spearman’s Rho *p* value	0.01 0.98	−0.2 0.5	0.34 0.24	0.24 0.39	0.2 0.50	0.29 0.31
	Reaction time phasic alert *n* = 15	Spearman’s Rho *p* value	0.16 0.59	0.05 0.85	0.34 0.24	0.37 0.17	0.29 0.31	0.29 0.32
	Standard deviation phasic alert *n* = 15	Spearman’s Rho *p* value	−0.19 0.50	−0.49 0.08	−0.22 0.45	0.16 0.58	−0.30 0.29	0.20 0.48
Executive function	Commission errors phasic alert *n* = 15	Spearman’s Rho *p* value	−0.12 0.68	−0.11 0.70	−0.33 0.25	0.04 0.89	0.03 0.92	−0.05 0.86
	Reaction time Stroop test *n* = 11	Spearman’s Rho *p* value	0.28 0.37	−0.01 0.97	0.15 0.64	0.23 0.45	−0.12 0.71	0.24 0.46
	Standard deviation Stroop *n* = 11	Spearman’s Rho *p* value	0.10 0.75	0.09 0.79	0.03 0.93	0.11 0.72	0.07 0.83	0.05 0.87
	Tower of London *n* = 11	Spearman’s Rho *p* value	−0.30 0.4	0.07 0.84	−0.21 0.55	−0.87 <0.001 *	−0.58 0.08	−0.51 0.13

Abbreviations: SWI = Spike Wave Index, SWF = Spike Wave Frequency. * Statistically significant correlations after multiple comparisons Bonferroni’s correction: *p*_corr_ < 0.05.

## Data Availability

The data presented in this study are available on request from the corresponding author. The data are not publicly available due to practical reasons.
